# Epidemiologic Status of Picobirnavirus in India, A Less Explored Viral Disease

**DOI:** 10.2174/1874357901812010099

**Published:** 2018-08-31

**Authors:** Yashpal Singh Malik, Shubhankar Sircar, Sharad Saurabh, Jobin Jose Kattoor, Rashmi Singh, Balasubramanian Ganesh, Souvik Ghosh, Kuldeep Dhama, Raj Kumar Singh

**Affiliations:** 1 ICAR-Indian Veterinary Research Institute, Izatnagar, Bareilly 243122, Uttar Pradesh, India; 2 College of Veterinary Sciences, DUVASU, Mathura, Uttar Pradesh – 281001, India; 3 Indian Council of Medical Research -National Institute of Epidemiology, R-127; 2nd Main Road, TNHB Layout, Ayapakkam, Chennai - 600 077, India; 4 One Health Center for Zoonoses and Tropical Veterinary Medicine, Ross University School of Veterinary Medicine, P. O. Box 334, Basseterre, St. Kitts, West Indies

**Keywords:** Picobirnavirus, Diagnosis, Epidemiology, Genogroup, RdRp phyloanalysis, RT-PCR, India

## Abstract

Since the unexpected discovery of picobirnaviruses (PBV) in 1988, they have been reported in many animals including mammals and birds, which comprises both terrestrial and marine species. Due to their divergent characteristics to other viral taxa they are classified into a new family *Picobirnaviridae*. Although their pathogenicity and role in causing diarrhea still remains a question since they have been discovered in symptomatic and asymptomatic cases both. Recent studies employing state-of-art molecular tools have described their presence in various clinical samples, like stool samples from different mammals and birds, respiratory tracts of pigs and humans, sewage water, different foods, *etc*. Furthermore, their epidemiological status from different parts of the world in different hosts has also increased. Due to their diverse host and irregular host pattern their role in causing diarrhea remains alien. The heterogeneity nature can be ascribed to segmented genome of PBV, which renders them prone to continuous reassortment. Studies have been hampered on PBVs due to their non-adaptability to cell culture system. Here, we describe the molecular epidemiological data on PBVs in India and discusses the overall status of surveillance studies carried out till date in India.

## 1 Introduction

Picobirnavirus (PBV) is connected with acute gastroenteritis cases in humans as well as animals. Although they were identified as birna-like viruses in 1988, its pathobiology is still uncertain. Further, viruses of this group remained in unclaimed virus family until 2009 when PBVs were recognized distinctly as members of the family *Picobirnaviridae* possessing double stranded bi-segmented RNA genome with human picobirnavirus as prototype species. Its genome is approximately 4.2 kbp, where the large segment, also known as genomic segment 1, is of 2.2-2.7 kbp and encodes the capsid protein, while another segment, also known as genomic segment 2, is of 1.2-1.9 kbp size and encodes the RNA dependent RNA polymerase (RdRp). The virion is non-enveloped, small, spherical, 33-41 nm in diameter and consists of a simple core capsid with a distinctive icosahedral arrangement [1].

Information gathered as of now on the epidemiology of PBVs rely primarily on the detection of viral dsRNA by Polyacrylamide Gel Electrophoresis (PAGE), electron microscopy or reverse transcription PCR (RT-PCR) detection assay based on two pairs of primers targeting the genomic segment 2 of PBV strains *viz*.; “4-GA-91” and “1-CHN-97” isolated in the USA and China, respectively [2]. PBVs are classified into genogroup I (prototype strain: GI/PBV/human/China/1-CHN-97/1997) and II (prototype strain: GII/PBV/human/USA/4-GA-91/1991). Based on the migration pattern of the bi-segmented dsRNA, PBV appears either with large genome profile or small genome profile. Likewise, based on differences in the genomic sequences of segment 2, PBV is further classified into genogroups I, II, non-I and non-II. Hitherto reports indicate the presence of divergent PBVs pointing towards the emergence of putative new genogroups in humans as well as animals [3, 4]. Culture of PBVs *in vitro* has not yielded any positive signal yet and there exit no animal model to study the pathobiology of virus infection, as well. Of the note, PBV has not been established yet as an etiological agent of diarrhea. Rather these are more often isolated as co-infecting agents with a number of diarrheal causes indicating their role in synergistic effect in association with the primary enteric cause. PBVs have been shown to cause gastroenteritis in immune-compromised individuals suggesting that immune status of the host plays an important role in establishing PBV infection as an opportunistic pathogen [5-8].

### 1.1 Epidemiology of Picobirnavirus

Picobirnaviruses were initially discovered by the finding of two nucleic acid segments through PAGE during investigations of acute gastroenteritis in children and from feces of black-footed pigmy rice rats [9, 10]. The virus has subsequently been detected in faecal samples from domestic and wild animal species, including rats, giant anteaters, guinea pigs, rabbits, chickens, pigs, calves, foals, cat and dogs [4, 9-34]. Epidemiological studies on PBVs in India are still in its early stages, with limited reports only. Here, we converse with the epidemiological studies carried out as on date in India on PBVs and present the current knowledge of PBVs in different hosts emphasizing the Indian perspective.

### 1.2 Human Picobirnaviruses

The actual journey of PBV discovery started in 1988, when Pereira and colleagues described a novel virus in human faeces serendipitously [9]. Subsequently, in 1995 PBVs were detected as co-infecting agents with Cryptosporidium in human faeces [15] and the virus was characterized by two extraction methods i.e. phenol/chloroform and guanidinium thiocyanate (GTC)/silica method followed by electron microscopy to know its physical properties [35]. In 2000, two prototype strains for GG-I (1-CHN-97) and GG-II (4-GA-91) were cloned and sequenced, obtained from human HIV infected patient and non-HIV infected patient from China and USA respectively [2]. Their presence has been reported in human faeces from Argentina [5, 8, 36, 37], Brazil [38], USA [7, 39, 40], Venezuela [41, 42], Hungary [43], Italy [44], Netherlands [45], Russia [46], Thailand [47] and Australia [48] that advocates circulation of the virus in several developed and developing countries of the world.

The available reports from India on PBVs in humans specify one geographical location *i.e*. Kolkata [49], the eastern part of the country. It was first reported in faecal specimens of children from a slum community [49], where out of the 56 diarrheic and 607 non-diarrheic samples screened, around 3.57% and 1.64% samples were found positive for PBV, respectively. Among the positive faecal specimens, both large (n=11) and small (n=1) genome pattern were observed in the PAGE analysis. Subsequently, from the same region, sporadic occurrence of this virus in humans was reported from 1999 to 2003 with detection of 21 (2.47%) positive out of 850 tested [50].All the positive samples were mostly taken from children which were showing acute watery diarrhea. These two reports showed the presence of both short genome profile as well as large genome profile PBV strains in India. Additionally, both reports showed diversity in human PBVs. In 2007, a report confirmed PBV infection in 2.06% of samples collected from children aged below 5 years [51]. Majority of the PBVs however, showed large genomic profile (n=22) as compared to the virus with small profile (n=1). These diverse sequence heterogeneity among the human PBVs belongto both GG-I and GG-II.

On molecular characterization of PBV positive samples from India in different studies, it was evident that PBV genogroup I is predominant in humans. Initial report of PBV in India, confirmed that genogroup I was more in asymptomatic (n=4) and symptomatic (n=1) cases and genogroup II was only diarrheic case [49]. Whereas, the subsequent study showed almost equal distribution of genogroup I (n=6) and genogroup II (n=5) but the study performed in 2007 showed higher prevalence of genogroup I (13/23) than genogroup II [50]. Although the presence of genogroup I as mixed infection was also reported from a diarrheic sample PBV/Human/INDIA/GPBV6/2007, in which 4 strains were from genogroup I and one strain from genogroup II was observed, suggesting intricate genetic reassortment and recombination episodes within the human PBV population [52]. Further, based on the sequence analysis and phylogenetic studies of several human PBV of genogroup I origin, it was found that they are closely related to the genogroup I strains of porcine origin which were earlier reported from Hungary, Venezuela and Argentina. This report also pointed towards the possibility of viral zoonoses from different animal species to human [52]. The presence of PBV as co-infection was also reported in the Indian studies discussed above, where PBV was associated with rotavirus (n = 3) or astrovirus (n = 3) or both (n=1) [49, 50] and Salmonella spp. (n = 1) [50]. However, no co-infection with norovirus, sapovirus or adenovirus or with parasites (Cryptosporidium spp., Giardia spp., Entamoeba spp., helminths) or bacteria (Vibrio spp., Shigella spp., Escherichia coli) was seen.

### 1.3 Bovine Picobirnaviruses

Though first report of PBV infection in bovines emerged from Russia in the year 1989 [22], reports on their prevalence remained obscured for a long time. PBVs were found in neonatal calves as sub-clinical infections along with Cryptosporidium parvum [53]. In the year 2003, Buzinaro and colleagues while determining the incidence of group A rotavirus (RVA) in dairy herds of Sao Paulo Brazil came to know the existence of this bi-segmented virus in four samples [23]. In 2003, Novikova and co-workers discovered this virus from faeces of a calf along with 3 cases of diarrhea in children in Russia [46]. A recent study in the year 2016 in Brazil reported small genome profile and the first sequence report from the American continent in bovine host [29].

The first detection of bovine PBV from India is attributed to the unanticipated discovery of this bi-segmented dsRNA virus strain, RUBV-P, in a one month old diarrheic calf. Molecular characterization of the strain RUBV-P for its full length gene segment 2 revealed distant genetic relatedness to other genogroup I PBVs of other animal species. Due to lack of sequence availability it was not possible to assess the genetic heterogeneity among bovine PBVs [24]. Subsequently, in one of our study carried out during 2007-2010 from subtropical (central India) and western Himalayan area of Uttarakhand, 136 faecal samples from buffalo (n=122) and cow calves (n=14) were screened for PBV. Among the five PBV samples identified, three were from buffalo calves and one from cow calf exhibiting acute diarrhea, while one sample was from a non-diarrheic buffalo calf. This report of PBV infection in diarrheic calves was based on RNA-PAGE analysis therefore sequence based assessment cannot be established with these samples [25]. Later on, we detected a buffalo PBV isolate from Maharashtra, India (PBV-B18) of genogroup I exhibiting very less homology of 44.5% and 45.1% at nucleotide and amino acid levels, respectively, with the prototype human PBV strain of GG I (1-CHN-97/AF246939). To note, this buffalo PBV isolate (B18) showed less homology with the previously reported bovine PBV strain from India (RUBV-P/GG-I/2005/GQ221268) suggesting this unique PBV isolate may represent an emerging heterogeneous group of virus with a new lineage [26]. In 2013, Mondal and co-workers also reported the presence of PBV infection in diarrheic buffalo and cattle calves along with co-infection of rotavirus though RNA-PAGE analysis [27]. The same author described the presence of GG-I type PBV in diarrheic calves from West Bengal, India [28]. These reports of PBV infection in diarrheic buffalo calves expanded our knowledge regarding the host range and their geographical distribution. In March 2012, a surveillance study on enteric viruses reported a unique bovine PBV isolate (HP-7) from diarrheic calves which carried dual infection of GG-I and GG-II genogroup [54]. Upon sequence analysis, it was found that HP-7-I was closer to Hungarian human PBV (Accession number AJ504796) and Brazilian canine PBV (Accession number FJ164032) isolates respectively. Whereas, HP-7-II was alike Indian human PBV (Accession number AB517738). This report supported the fact of finding frequent mixed infection of PBVs comprising both genotypes in humans and porcine population [52, 55].

### 1.4 Porcine Picobirnaviruses

The earliest report of PBV infection in pigs dates back in 1989 when this dsRNA virus was discovered by polyacrylamide gel electrophoresis in Brazil [17]. Bi-segmented banding pattern confirmed the presence of PBV infection which was also observed during the investigation of intestinal content from human, pigmy rice rats and guinea pig. Consecutively, in successive years detection of this bi-segmented pathogen was also observed in different diagnostic labs of UK [18] followed by various Latin American studies on porcine PBV in Venezuela [19, 56, 57], Brazil [58], Argentina [59, 60] which has also reported this virus in due course of detecting various other enteric pathogens or working specifically for PBV diagnosis. A study in Hungary also elaborated the possibility of interspecies transmission among the porcine and human PBV strains [20]. The PBV isolates detected in Venezuela, Argentina and Hungary were closely related to human GG-I PBVs [20, 56, 60]. In 2011, Smits and colleagues reported the PBV infection in respiratory tracts of pigs, signifying their role as respiratory pathogens [55]. While working on a Porcine PBV strain 221/04-16, Banyai and co-workers also demonstrated that due to high degree of insertions/deletions in the PBV genome are responsible for short and long type of genome segments resulting in sequence length heterogeneity [61]. These reports regarding the presence of multiple PBV strains present in pigs also emerged from China in the year 2014 [62]. Reports emerged in Thailand where diarrheic and non-diarrheic piglets were found to possess the PBV infection along with co-infection of group A rotavirus (RVA) and bocavirus (BoV), all PBV strains were belonged to GG-I genogroup and phylogenetically they were close to Chinese PBV strains [63].

The first detection and molecular characterization of porcine PBV (BG-Por-2/2010 and BG-Por-7/ 2010) was reported from a piggery located in an urban slum at Kolkata, India [64]. The phylogenetic analysis revealed genetic similarity to different porcine and human GG-I PBVs from different geographical regions. In 2016, Porcine PBV infection was reported from North Eastern Region of India with strains having varied diversity and were not found to be closely associated with any other Indian isolates of PBVs so far [21]. The study also claims that PBV infections are higher in acute summer and winter seasons compared to spring and autumn seasons with high prevalence recorded in cross breed pigs in contrast to local breeds.

### 1.5 Picobirnaviruses in Other Species

With the availability of high end instruments and state-of-art techniques, PBVs have been detected in several un-common animal species throughout the world. In 2015, researchers explored the virome of 458 rhesus macaques collected from different geographical areas of neighboring country Bangladesh [65]. They applied the combination of consensus PCR (cPCR) along with High Throughput Sequencing (HTS) to characterize 184 viruses from 14 virus families. Out of them all, 120 were PBV of the 184 viruses found in these animals which showed the presence of this bipartite virus in the population of nearby primate population. Moreover these free ranging macaques are usually found near the human settlements and therefore the risk of zoonoses cannot be ruled out. Earlier, high incidence of PBV infection in asymptomatic primates was also observed in Argentina where individually caged orangutans were diagnosed with persistent PBV infections over the span of three years [66]. Application of metagenomics sometime reveals unknown pathogens in unusual hosts and gives a comprehensive picture regarding genetic map of those pathogens, similar study was performed in Portugal in 2015 where PBV infection was found in wolves [67]. This study also described the reassortment events going among PBVs in which identical capsid segments together with diverse RdRp segments were found.

Alike, a PBV strain, PBV/Horse/India/BG-Eq-3/2010, was identified in the faeces of a 10 month old weaned female foal with diarrhoea in January 2010 from Kolkata, India [31]. Surprisingly, sequence comparison and phylogenetic analysis revealed close genetic relatedness to a human genogroup I PBV strain (Hu/GPBV1) detected earlier from the same part of India.

Using RT-PCR assay, PBV has been detected in 2.4% (3/125) and 3% (3/100) diarrheic kids and lambs fecal samples, respectively, from July, 2013 – February 2014 in Northern parts of India. Three PBV isolates, each from caprine and ovine species were typed as genogroup I (GGI) while two ovine isolates were typed as genogroup II (GGII). Even in one of the ovine samples both genogroups were found as a mixed infection (Unpublished data).

PBVs have also been defined to be present as persistent infection in captive animal species which expanded our knowledge regarding the host range of this virus [68]. Although the diagnosis method applied in that study was RNA-PAGE which has lower sensitivity then RT-PCR therefore the number of PBV positive species could have been more if RT-PCR assay would have been applied. Similarly, PBV infection in captive feline species was also described in 2012 in Uruguay where four different feline species were found to possess the PBV infection [69]. In 2009, a report emerged from Brazil in which further investigation on captive species like Wistor rats, snakes and domestic dogs was carried out to characterize PBV from these species and they applied the RT-PCR diagnosis method to diagnose the infection [70]. This study shed more light on co-circulation of PBV in different hosts and identified snakes as a new host for PBVs. Following reports of PBV infection in different hosts Woo and colleagues discovered a novel PBV in feces of Calfornia Sea Lions and prosed a new species as otarine PBV for which they published the whole genome [71]. These reports contributed in the understanding that PBV infections are persistent in symptomatic as well as asymptomatic animals which includes both mammals and birds [72]. Studies on domesticated birds like turkey and broilers have also reported the diagnosis of PBV infection in USA and Brazil [73, 74]. In 2014, a thorough NGS study on camel feces revealed huge abundance of PBV infection co-infecting the camel population along with other viral pathogens [75]. Keeping in mind the findings of Woo and colleagues, in 2016 we diagnosed PBV infection in captive camel population of Gujarat state where 93.10% of samples were found positive with no diarrheic symptoms (Unpublished data). Likewise, a comprehensive molecular epidemiological study was conducted in several mammalian species of terrestrial and marine habitat in Hong Kong revealed high diversity among different mammals [76]. These studies expanded our knowledge regarding the increase in host range of PBV’s and their greater susceptibility to domesticated, terrestrial as well as marine mammalian species. The risk of species jumping among different PBV strains is an alarming condition which can be addressed by continuous surveillance of this virus in different mammalian and avian species.

### 1.6 Evolutionary Analysis of Picobirnaviruses

The total number of nucleotide submission of PBV from different species are very few from India (n=87) (Table **1**). Out of the 87 sequences only one sequence is having complete information of RdRp gene (Segment 2).

**Fig. (1):**
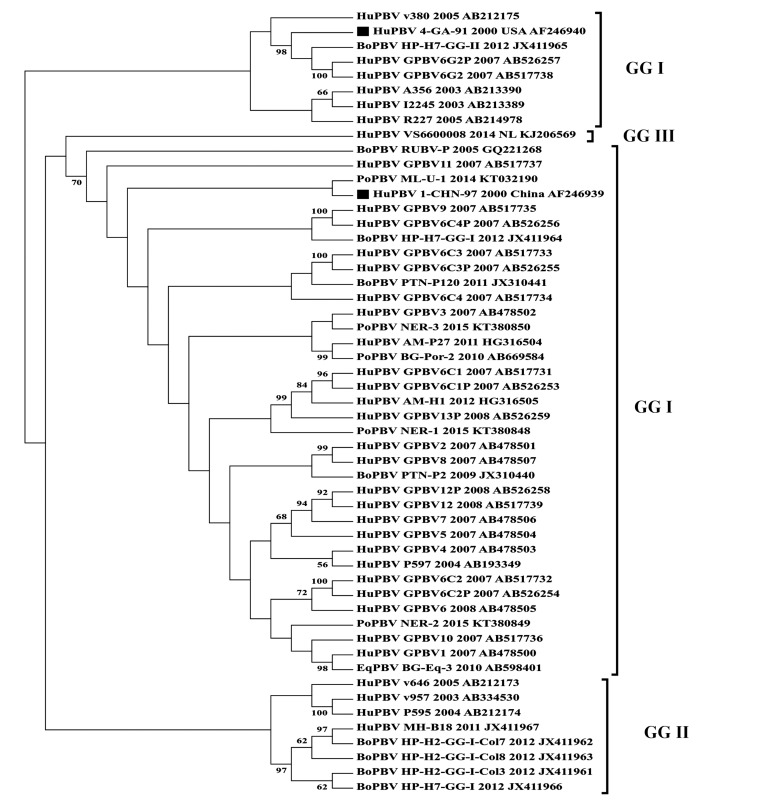
Phylogram display genetic diversity between different Picobirnavirus populations in India.

Sequences from different species of Indian PBV population were phylogenetically analyzed using the MEGA 6.0 software applying the General Time Reversible model along with Gamma distribution which was used to model evolutionary rate differences among sites [77]. The analysis included 53 sequences comprising the reference sequences of PBV for GG-I and GG-II (Fig. **1**). Huge diversity was observed within the Indian PBV sequences when they were phylogenetically analyzed suggesting species jumping with no specific host among the Indian PBV population. Although the available GG-I and GG-II sequences had formed a separate clade (Fig. **1**). The bipartite nature of PBVs renders them as possible candidates for further recombination and reassortment among the different isolates [20, 43]. A recent report has also confirmed and speculated that animal and human PBV strains can re-assort independently [67].

**Fig. (2):**
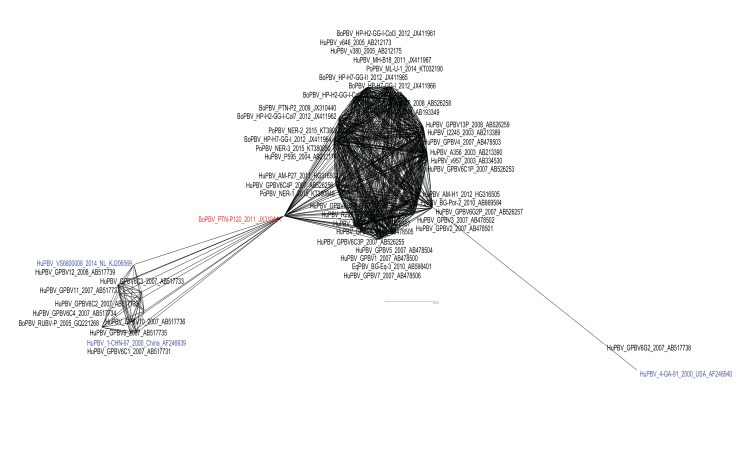
Minimum spanning network exhibiting diversity between Indian Picobirnavirus isolates.

Further, to confirm the species diversity among Indian PBV isolates, the dataset was analyzed using the SplitsTree4 software package [78]. The Minimum Spanning Network applied to the dataset bifurcated the isolates into two major Indian PBV populations which shows a bigger network comprising of human, bovine, porcine and equine isolates reported till date whereas, the smaller population comprises majorly of human PBV isolates with only one bovine PBV strain RUBV-P also clustering along with these human strains (Fig. **2**). The recently proposed GG-III genogroup isolate from Netherlands (VS6600008) also clustered within this bunch of human origin PBV isolates.

Interestingly, in this analysis a bovine PBV (PTN-P120) isolate emerged as a pivotal strain from where two major clusters were seemed to have emerged in the Indian PBV population. The reference strain for GG-II PBV (4-GA-91) was again distantly placed beside the Indian human PBV strain (GPBV6G2) from Kolkata which also belong to GG-II genogroup. Rest all other strains pertaining to GG-I and GG-II were clustered inside the major constellation of Indian PBV isolates. The analysis suggested that these all isolates are resultant of species jumping, where GG-I and GG-II sequences were seemed to have co-evolved with each other having no clear ancestor among them.

Nevertheless, due to limited epidemiological data available on PBVs in Indian human and animal population we can’t approach to a concrete conclusion regarding the evolution, host-virus relationship and the infection pattern of PBV in Indian perspective.

## CONCLUSION AND FUTURE PROSPECTS

Since their emergence in 1988, picobirnaviruses have established themselves as cause of great concern worldwide. However, PBVs have formally not yet been associated with diarrhea. An adequate cell culture system or an animal model has not been established till date, which implicates several drawbacks in understanding the biology and pathogenicity of PBVs. They have shown association with gastroenteritis and respiratory infections. The grave notch is that they can inflict wide range of susceptible hosts. The genetic relatedness between human and animal PBV warrant further studies on zoonotic potential. Therefore, continued surveillance is needed to assess the emergence of new PBV in different host, its origin, diversity and public health implications. Moreover, the tropism of this virus is still in question and its relevance as a major causative agent for neonatal or adult diarrhea is still a matter of speculations. The persistence of PBV and its diagnosis in a host with or without the signs of diarrhea always contradicts the different studies which claim it as notable cause of diarrhea. Also, the detection of PBVs from sewage and surface water, indicates that the virus continues to exist in various diverse environmental conditions and also being shed from asymptomatic carrier host animals. A clear picture of the virus-host-environment is to be studied under the “One Health” concept. With only few records of sequence data available on the GenBank database and negligible number of complete gene information from India the need of active surveillance based research becomes important in future. Improved management practices should be adopted on animal farms for prevention and control of picobirnavirus infection.

## AUTHOR CONTRIBUTIONS

All the authors substantially contributed to the conception, design, analysis and interpretation of data, drafting and revising, updation, checking and approving final version of manuscript. We all agree to be accountability for the content of this manuscript.

## Figures and Tables

**Table 1:** Total number of nucleotide submissions in GenBank for picobirnavirus from India.

Sl. No.	Species	No. of Sequences	Partial	Full
1	Human	33	33	00
2	Bovine	10	09	01*
3	Porcine	36	36	00
4	Equine	01	01	00
5	Caprine	03	03	00
6	Ovine	04	04	00
Total		87	86	01

* Only complete RdRp gene (Segment 2).
